# G-quadruplex topologies determine the functional outcome of guanine-rich bioactive oligonucleotides

**DOI:** 10.1093/nar/gkaf590

**Published:** 2025-06-28

**Authors:** Prakash Kharel, Nupur Bhatter, Safiyah Zubair, Shawn M Lyons, Paul J Anderson, Pavel Ivanov

**Affiliations:** Division of Rheumatology, Inflammation, and Immunity, Department of Medicine, Brigham and Women’s Hospital, Harvard Medical School, Boston, MA 02115, United States; Division of Rheumatology, Inflammation, and Immunity, Department of Medicine, Brigham and Women’s Hospital, Harvard Medical School, Boston, MA 02115, United States; Division of Rheumatology, Inflammation, and Immunity, Department of Medicine, Brigham and Women’s Hospital, Harvard Medical School, Boston, MA 02115, United States; Department of Biochemistry and Cell Biology, School of Medicine, Boston University, Boston, MA 02118, United States; Division of Rheumatology, Inflammation, and Immunity, Department of Medicine, Brigham and Women’s Hospital, Harvard Medical School, Boston, MA 02115, United States; Division of Rheumatology, Inflammation, and Immunity, Department of Medicine, Brigham and Women’s Hospital, Harvard Medical School, Boston, MA 02115, United States

## Abstract

Guanine-rich nucleic acid sequences can exert sequence- and/or structure-specific activities to influence biological and pathobiological cellular processes. As such, it has been reported that different G-rich oligonucleotides (both DNA and RNA) can have cytotoxic as well as cytoprotective effects on the cells. However, the mechanisms of such a biological outcome are unclear. Here, we report that G-rich DNA oligonucleotides (ODNs) that can form four-stranded secondary structures called G-quadruplexes (G4s) have a topology-dependent biological outcome. Using different biochemical, biophysical, and cellular approaches, we demonstrate that only the parallel topology G4-forming ODNs can repress eukaryotic messenger RNA (mRNA) translation by directly interacting with eukaryotic translation initiation protein 1 (EIF4G1), while the anti-parallel topology G4s do not have inhibitory effect on mRNA translation. These results directly connect the G4 topological differences within ODNs to differential functional impacts in mRNA translation *intrans*. Our study provides the foundation for the rational design of G-rich oligonucleotides for a desired therapeutic outcome.

## Graphical Abstract

**Graphical Abstract:**
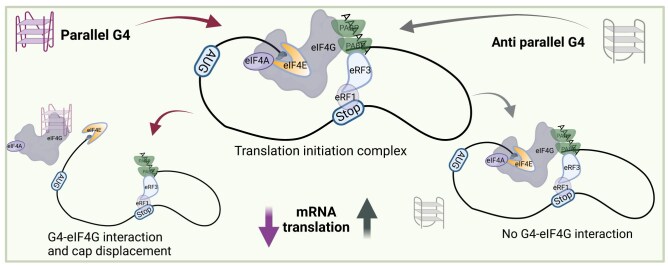

## Introduction

Oligonucleotide (ON)-based therapeutic molecules can regulate gene expression via several different mechanisms such as RNA interference, target degradation by RNase H-mediated cleavage, splicing modulation, gene editing, and gene activation [1]. Among different categories of therapeutic ONs, guanine (G)-rich sequences bring an additional dimension due to their unique structural and functional properties [2, 3]. G-rich oligodeoxynucleotides (ODNs) and ONs have been reported to exhibit a wide range of biological effects, encompassing cytotoxic [4, 5], anti-cell proliferative [6], and cytoprotective activities [7]. The anti-proliferative and pro-apoptotic effects of G-rich ODNs/ONs have been observed in various cell types, including vascular smooth muscle cells and numerous cell lines derived from solid tumors, leukemias, and lymphomas. Importantly, while most common nucleic acid-based therapeutic strategies depend upon their antisense properties (complementarity of the drug–target pair), G-rich ODNs/ONs are known to exhibit unique non-antisense (e.g. specific protein binding) properties when delivered to target cells [8]. In addition, the non-antisense antiviral effects of G-rich ODNs were first noted almost three decades ago [9].

As such, so-called G-rich T-oligos (DNA oligos homologous to the telomere 3′ overhang region, e.g. pGTTAGGGTTAG) have been extensively studied as potent DNA damage mimics in normal cells evidenced by their ability to induce transient cell cycle arrest in normal human cells of many lineages [5]. Importantly, G-rich T-oligos were shown to induce more potent and selective cytotoxicity on malignant cells compared to their normal counterparts and so they have been long touted as potential anticancer drugs [10, 11]. For some ODNs/ONs, *in vivo* anti-proliferative effects were also demonstrated in tumor-bearing mice. Furthermore, G-rich ODNs/ONs containing several backbone modifications can bring a diverse array of biological effects upon their cellular delivery, e.g. inhibition of cell proliferation, induction of cell death, changes in cellular adhesion, inhibition of protein aggregation, and antiviral activity [12].

**Figure 1.**
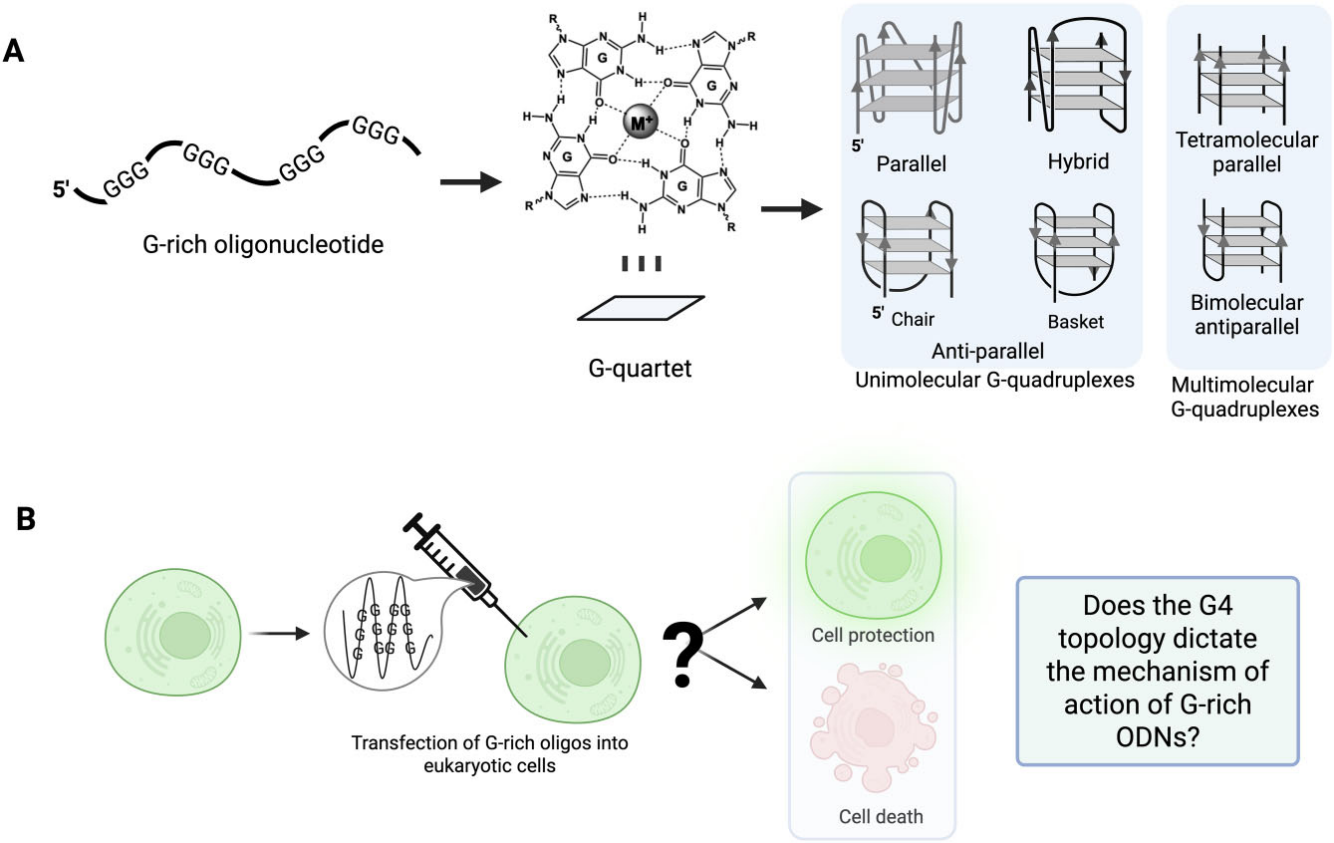
G4 structures and different outcomes of therapeutic G-rich ODNs. (**A**) G-rich ODNs can fold into different kinds of G4s depending upon their ionic and sequence environments. (**B**) Traditionally, G-rich ODNs have been used in different biological experiments as pro-drug molecules and showed widely different outcomes.

Somel bioactive G-rich ODNs/ONs can fold into four-stranded secondary structures called G-quadruplexes (G4s) via stacking of square planar G-quartets in a cation-assisted manner (Fig. 1A) [6, 13, 14]. Depending upon the nature of their sequence and microenvironment, G-rich nucleic acid sequences can also fold into different G4 topologies [15]. Both Watson–Crick and Hoogsteen sides of G base are involved in H-bonding in the formation of G4s [14]. While almost all reported RNA G4s (rG4s) and many DNA G4s (dG4s) have parallel topologies, a conformational switch around glycosidic bond resulting in *syn-*conformation (compared to the sterically favorable *anti-*conformation) allows the formation of anti-parallel loops and hence anti-parallel as well as mixed topology (hybrid) G4s in DNA [16]. Additionally, while most of the reported genomic and transcriptomic G4s are unimolecular, bimolecular and tetramolecular G4s are common, particularly at the ON level. G4s in both DNA and RNA possess a wide range of biological functions *in cis* depending upon the context of their presence and their surrounding environment [13]. Several factors such as conformational polymorphism, loop permutations [17], spatial restriction [18], and stability variations [19] endow G4s with diverse functional applications as aptamers, fluorescence probes and nanodevices, biosensors, and drugs [20]. In some cases, G4 ODNs and ONs facilitate their function by directly interacting with G4-binding cellular proteins (G4BPs) [21], thus leading to cytotoxicity [6, 22] or cytoprotective [7] effects. Alternatively, G-rich ODNs/ONs can bind to specific cellular proteins independent of G4 secondary structure [23] to alter cell proliferation.

Binding of various G4s to multiple proteins is well described [24–26]. In some cases, binding of G4s to specific proteins can facilitate their phase transition, e.g. from more liquid to more solid, promoting their aggregation [27]. Such aggregations are commonly associated with human pathological processes, e.g. neurodegeneration. Specific examples of these include interaction of rG4s with Tau, an intrinsically disordered protein that plays key roles in the pathologies like Alzheimer’s disease, frontotemporal dementia and other tauopathies. Various rG4s promote Tau phase transition under near-physiological conditions *in vitro* [28]. Likewise, in Parkinson’s disease, rG4s accelerate sol–gel phase transition α-synuclein, a principal player in this disease [29]. Interestingly, unbiased *in vitro* screen of single-stranded ODNs on their intrinsic abilities to act as a chaperone for specific protein substrates identified a bias in the ability of anti-parallel quadruplexes to increase protein aggregation and possess no chaperone activity. In contrast, hybrid and parallel G4s seem to act as protein chaperons [30]. Whether G4 ODNs can actually exert their effects in a topology-dependent manner in the context of biological processes, beyond its biophysical effects on specific substrates *in vitro*, is an open question.

While most of the reported cellular G4s act *incis*, the G4s formed by smaller nucleic acids including therapeutic ODNs/ONs mostly act *in trans* and obviously have a potential to influence multiple targets. Even though G-rich ODNs/ONs have been reported to demonstrate unique biological properties, it is not clear what makes some of the G-rich ODNs/ONs act differently than the others (Fig. 1B). In this study, we analyzed the properties of several G-rich ODNs reported in literature for their diverse biological activities and discovered that such ODNs are translationally active (active mRNA translation repressors) only when they fold into parallel topology G4s (p-G4s). Importantly, our results demonstrate that a switch in G4 conformation from anti-parallel G4 (ap-G4) to p-G4 can revert their biological functions. Additionally, we identify molecular mechanism by which G-rich ODNs impact cell biology by targeting the global translation machinery in the human cells.

## Materials and methods

### ODNs/ONs

**Figure 2.**
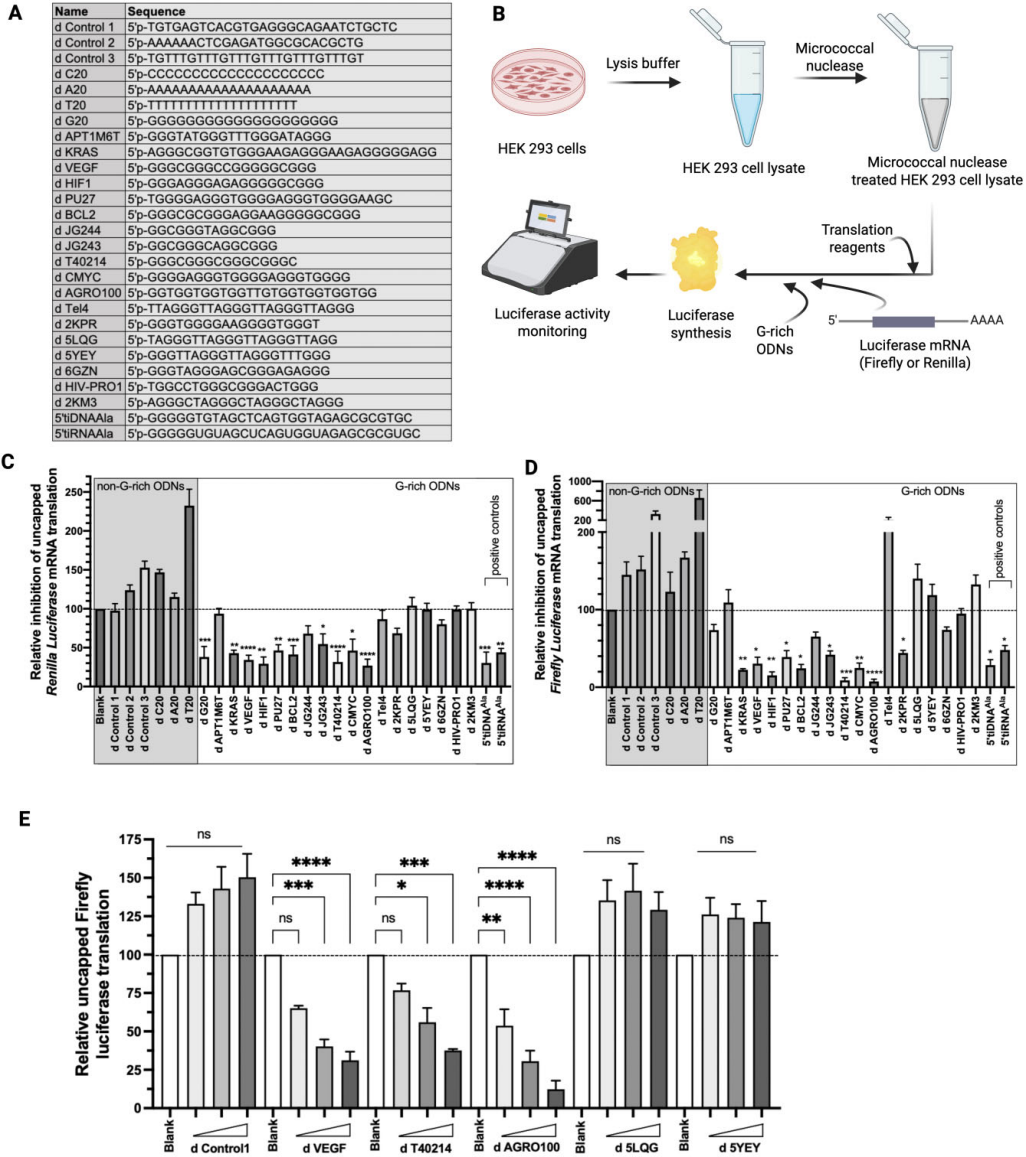
G4-forming G-rich ODNs have distinct impact on the translation of reporter messenger RNAs (mRNAs). (**A**) Sequences of G-rich and non-G-rich ODNs used in this study. More details on the characterization/application/reference of these ODNs are presented in Supplementary Table S1. (**B**) Schematic representation of the experimental procedure to test the bioactivities of tested ODNs. (**C**) Impact of different G-rich ODNs in the translation of uncapped Renilla luciferase (RL) mRNA in HEK293 cells-derived lysate. ODN/ON concentration was 10 μM, except for blank. (**D**) Impact of different G-rich ODNs in the translation of uncapped Firefly luciferase (FL) mRNA in HEK293 cells-derived lysate. ODN/ON concentration was 10 μM, except for blank. (**E**) Concentration-dependent effect of bioactive G-rich ODNs in the translation of uncapped FL translation. Different concentrations of G-rich ODNs used: d Control1, d 5LQG, and d 5YEY—10, 20, and 30 μM; d VEGF, d T40214, and d AGRO100—2.5, 5.0, and 10 μM. The bars represent ± standard error of mean (SEM) of three independent biological experiments; statistical significance was tested by one-way ANOVA using water treatment as a blank control, multiple comparisons, and Dunnet correction, and *P*-values indicated only where there is a significant repression. *P< .5, **P< .05, ***P< .0005, and ****P< .00005.

All the ODNs and ONs were purchased from IDT. Stock solutions were made up to a concentration of 100 μM in nuclease-free water and stored at −80°C. The ONs used are listed in Fig. 2A and Supplementary Table S1.

### Cell culture and preparation of translation-competent mammalian extract

Human embryonic kidney 293 (HEK293) cells were maintained in Dulbecco’s modified Eagle’s medium (Gibco) containing 10% fetal bovine serum with 1% penicillin–streptomycin. After reaching 80%–90% confluency, HEK293 cells were detached via trypsinization at 37°C for 2 min. Equal volume of DMEM was added to stop the trypsinization reaction and cells were pelleted at 5000 rpm for 2 min. The medium was removed, and cells were suspended in 1 ml of hypotonic lysis buffer (10 mM HEPES, pH 7.0, 10 mM potassium acetate, 0.5 mM magnesium acetate, 5 mM DTT, and one tablet of mini ethylenediaminetetraacetic acid-free protease inhibitor from Roche). The cells were transferred to a 1.5 ml-microcentrifuge tube and centrifuged at 1000 RCF at 4°C for 1 min. The supernatant was removed, and the cells were resuspended in chilled lysis buffer (volume of lysis buffer is same as volume of packed cells). Cells were tumbled at 4°C for 1 h followed by homogenization by passing through a 1-ml syringe 27G needle for 10–12 times. The resulting lysate was centrifuged at 14 000 RCF for 1 min and 40 μl of supernatant was aliquoted in 1.5 ml-microcentrifuge tubes. The tubes were snap frozen in dry ice followed by immediate storage at −80°C.

### Reporter mRNA preparation

The following reporter mRNAs were used in this study. Uncapped FL mRNA (Promega^®^, Cat# L4561) was directly used from the kit without further modification. Capped FL mRNA was prepared using Cellscript m7G capping kit (Cat# C-SCCS1710) as per manufacturer’s protocol. RL reporter mRNA was transcribed *in vitro* using Promega Ribomax T7 transcription kit (Cat# P1300) using SacII digested pHRL control plasmid. Capped RL mRNA was prepared using Cellscript^®^ m7G capping kit (Cat# C-SCCS1710) as per manufacturer’s protocol.

### *In vitro* translation

*In vitro* translation reaction in HEK293 cell extracts was performed as described in a previous report [31]. Briefly, a 10 μl reaction was set up with different amounts of respective ODNs with 100 ng of reporter mRNA, 1.25 mM of magnesium acetate, 150 mM potassium acetate, 1× translation buffer (1.6 mM HEPES, pH 7, 2 mM creatine phosphate, 0.01 μg/ml creatine kinase, 10 μM spermidine, and 10 μM l-amino acid mix, Promega^®^, Cat# L4461), 1 unit of RNase inhibitor (Promega^®^, Cat# N251B), and micrococcal nuclease (Thermo Fisher Cat# 88216) treated mammalian extract. The tubes were then incubated at 37°C for 1 h followed by luminescence reading. Depending upon the reporter mRNA used, 20%–50% of the reaction was mixed with either Firefly or Renilla substrate and luminescence was measured using Promega^®^ Glomax Explorer Machine.

### ODN/ON folding

ODNs/ONs were folded in 150 mM KCl or LiCl in T_10_E_0.1_ buffer (10 mM Tris–HCl, pH 7.4, and 0.1 mM EDTA) by heating at 95°C for 5 min and then slowly cooling to room temperature over ∼1 h period by letting the oligo tubes in heat block sit at room temperature.

### CD spectroscopy

JASCO J815 spectropolarimeter was used to collect circular dichroism (CD) spectra. The ONs were dissolved in 150 mM K^+^ in T_10_E_0.1_ buffer. Quartz cuvettes (1 mm path length) were used with sample volumes of 200 μl to achieve a sample concentration of 5 μM. Spectra were collected in the range between 200 and 320 nm at 20°C from three scans, and a buffer baseline was subtracted from each spectrum. CD was expressed as the difference in the molar absorption of the right-handed and left-handed circularly polarized light. An increased peak intensity of the oligo under K^+^ environment at 260–265 nm and a trough at 240 nm, which shows a reduced peak intensity under Li^+^ environment, suggest the formation of a p-G4, whereas a cation-responsive peak at around 295 nm indicates an antiparallel topology.

### Purification of recombinant protein

eIF4G1 (eukaryotic translation initiation factor 4G 1)-HEAT1 was expressed and purified as described previously [21]. eIF4G1-HEAT1-expressing plasmid, pET21-10XHis-Smt3-eIF4G (HEAT1), was transformed into Rosetta 2 pLysS *Escherichia coli* cells. Cells were grown at 37°C in Terrific media until the OD reached ∼0.8 and then induced with 500 μM isopropyl β-d-1-thiogalactopyranoside (IPTG) for 16 h at room temperature. Cells were pelleted. eIF4G1-HEAT1 was purified by disrupting cells by sonication in 20 mM Tris (pH 8.0) and 150 mM NaCl. Lysate was clarified by centrifugation (20 min at 21 000 × *g*). Lysate was incubated with 2 ml of Ni-NTA agarose (Invitrogen) for 2 h at 4°C. Beads were washed 1× with 20 mM Tris (pH 8.0), 150 mM NaCl, and 10 mM imidazole, then 1× with 20 mM Tris (pH 8.0), 500 mM NaCl, and 10 mM imidazole, and then 1× with 20 mM Tris (pH 8.0), 150 mM NaCl, and 10 mM imidazole before elution with 20 mM Tris (pH 8.0), 150 mM NaCl, and 250 mM imidazole. Eluted protein was dialyzed overnight into 10 mM sodium phosphate (pH 7.5), 300 mM NaCl, 2 mM β-mercaptoethanol, 5 mM MgCl_2_, and 5% glycerol. The following day, the protein was dialyzed against fresh buffer for an additional 4 h. Precipitate was cleared by centrifugation and supernatant was concentrated with Amicon Ultra-15 Centrifugal Filter Unit (NMWL 30 kDa). Finally, ATP was added to a final concentration of 1 mM.

### Electrophoretic mobility shift assays

Folded ODNs/ONs and proteins were mixed with increasing concentration of eIF4G1-HEAT1 in 10 mM sodium phosphate (pH 7.5), 300 mM NaCl, 2 mM β-mercaptoethanol, 5 mM MgCl_2_, 5% glycerol, and 1 mM ATP and incubated at room temperature for 15 min before loading onto 20% acrylamide gel (29:1 acrylamide:bis-acrylamide, 0.5× TBE). The gels were stained with SYBR Gold and scanned.

## Results

### G-rich ODNs differ in their ability to modulate mRNA translation repression

Previously, we demonstrated that several transfer RNA (tRNA)-derived stress-induced RNA molecules (tiRNAs) can repress eukaryotic mRNA translation [31–33]. Importantly, two of the translation-repressing tiRNAs contain 5′-**T**erminal **O**ligo**G**uanine (5′TOG) motif, which was shown to be specifically responsible for their repressing activity [21, 32, 34]. Furthermore, we and others demonstrated that several G-rich ODNs and ONs can impart different physiological outcomes in normal and stressed cells [7, 35]. Translation of mRNA in eukaryotic cells is strictly regulated to modulate protein synthesis during cellular homeostasis, development, and cellular stress [36]. Because we previously showed that tetramolecular G4-forming tiRNAs can repress rabbit reticulocyte (RRL)-based *in vitro* translation (IVT) of uncapped Firefly luciferase (FL) mRNA in RRL [32], we asked whether translation repression is the characteristic of all G4-forming ODNs/ONs.

To answer this question, we empirically picked several G-rich ODNs (Fig. 2A and Supplementary Table S1) that were already mentioned in the literature for their wide range of bioactivities, remarkably as contributing to cell death or survival. As schematically demonstrated in Fig. 2A, we performed IVT experiments in the presence of several G-rich ODNs and control non-G-rich ODNs along with two positive controls (namely, 5′tiRNA^Ala^ and 5′tiDNA^Ala^). Our ODN pool includes G-rich ODNs with wide variations, e.g. G-rich ODNs that start with the G stretches in the 5′-end and those that do not, ODNs that have several two G stretches separated by various non-G nucleotides, and ODNs that have several three and four G stretches. We prepared IVT lysates from HEK293 cells and analyzed the ability of these G-rich ODNs to inhibit the translation of different mRNA luciferase reporters in these systems (as schematically illustrated in Fig. 2B).

First, we performed analysis of Renilla luciferase (RL) mRNA translation in the presence of different ODNs (Fig. 2C). Quite interestingly, we observed that while there is a variable enhancing impact of non-G-rich control ODNs in RL mRNA translation, G-rich ODNs appeared to influence the RL translation in two separate ways. One group of G-rich ODNs repressed reporter translation, as we previously observed in case of tetramolecular assembly forming TOG motif containing 5′tiRNA^Ala^ and its DNA counterpart, namely 5′tiDNA^Ala^; while the other group of G-rich ODNs showed no significant impact on RL translation. Next, we asked whether this selective behavior of G-rich ODNs is expandable to the translation of other mRNAs. To answer this question, we performed identical experiments with FL mRNA (Fig. 2D). Interestingly, the set of G-rich ODNs that showed inhibitory effects in RL translation also repressed FL translation, while translationally inactive G-rich ODNs mostly remain inactive in both cases (Fig. 2C and D).

Furthermore, we expanded our investigation to study the impact of G-rich ODNs in the translation of capped RL and FL mRNAs in custom-made cellular IVT extracts. While the extent of translation repression from the translationally active G-rich ODNs is less effective under the similar translation condition, both sets of G-rich oligos follow the previously observed pattern, albeit more loosely (Supplementary Figs S1 and S2).

Next, we asked whether the translation repression activity of active G-rich ODNs is a concentration-dependent. To answer this, we analyzed uncapped FL translation in the presence of different concentrations of select candidates from the pools of both active and inactive G-rich ODNs (namely, d Control1 as a non-G-rich control; d VEGF, d T40214, and d AGRO100 as active G-rich ODN candidates; and d 5LQG and d 5YEY as inactive G-rich ODN candidates) (Fig. 2E). A clear dose-dependent effect was observed for active G-rich ODNs, while there was no translational impact by the inactive G-rich ODNs even at the higher molar concentrations.

### Bioactive ODNs form topologically different G4s than the inactive ODNs

**Figure 3.**
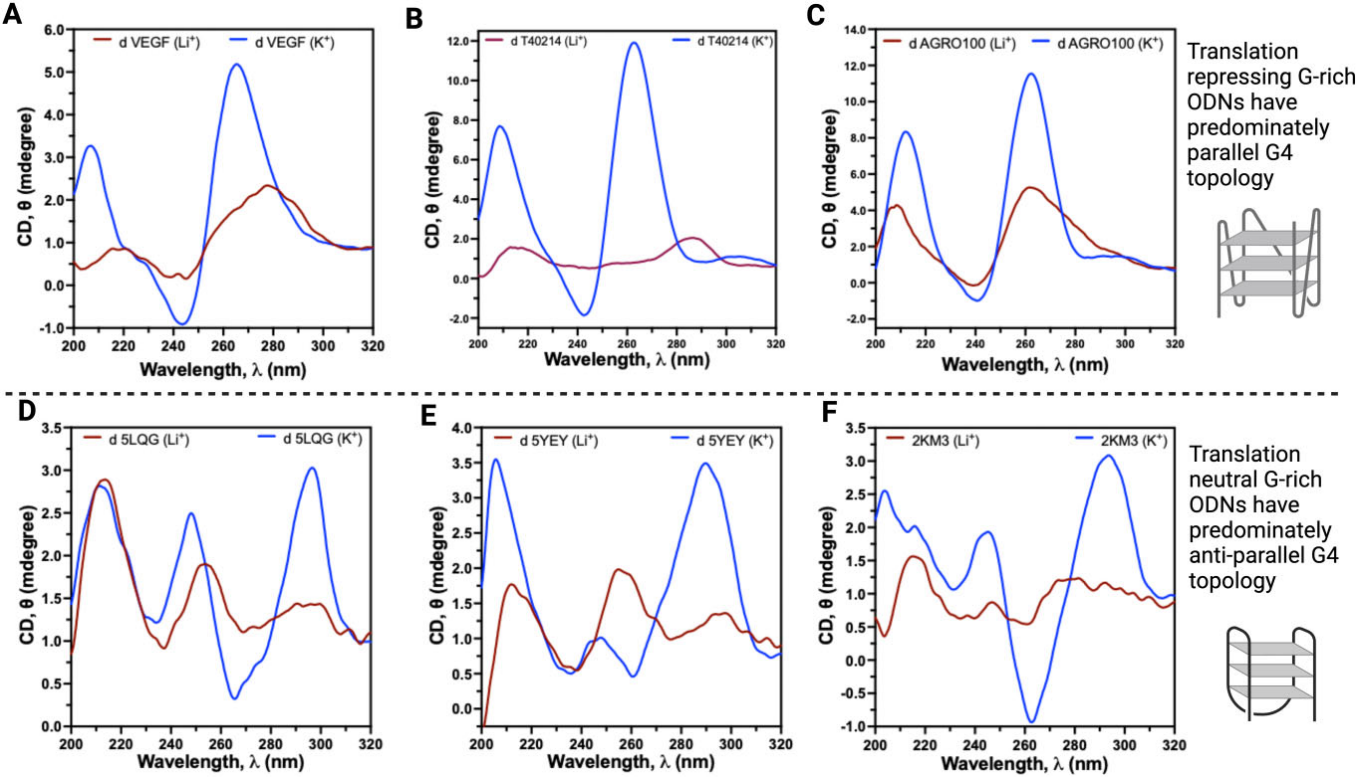
Translation-repressing G-rich ODNs fold into topologically different G4s compared to translationally inactive G-rich ODNs. (**A**–**C**) All translation-repressing G-rich ODNs fold into predominantly p-G4s, and (**D**–**F**) translationally inactive G-rich ODNs fold into ap-G4s. CD spectra of all the G-rich ODNs used in this study are presented in Supplementary Fig. S3.

Most of the bioactive G-rich ODNs discussed in the literature and all G-rich ODNs we picked for this study have a potential to form G4s. Yet they possess different bioactivities, and we reasoned that differences in G4 topology could be a contributing factor to their function. To test this hypothesis, we monitored the G4 folding behavior of G-rich ODNs using CD spectroscopy. In CD, a cation-responsive (higher intensity at G4 permissive K^+^ environment and lower at G4 non-permissive Li^+^ environment) positive peak at ∼263 nm and a trough at ∼240 nm are indicative of a p-G4 topology, while a peak at ∼295 nm indicates an ap-G4 topology. To test the G4 topologies shown by G-rich ODNs, they were folded in different buffers at 10 μM ON concentration and analyzed using CD spectroscopy. Interestingly, all the active (translation-repressing) G4 ODNs showed predominantly parallel topology, while those not showing translation repression activity showed predominantly anti-parallel topologies (Fig. 3A and Supplementary Fig. S3).

### Parallel to anti-parallel switch of G4s (without changing the base composition) rescues G4 ODNs’ bioactivity

Next, we asked whether the functional differences of p-G4 versus ap-G4 ODNs are truly due to their structural and topological variations and not because of the differences in the sequence composition. To test this, we modified translationally inactive ap-G4 ODNs to their RNA counterparts. Since the presence of 2′-OH group enforces parallel topology in rG4s [37] and almost all reported rG4s form pG4s, we reasoned that DNA to RNA conversion of G-rich ODNs should lead to p-G4s. If the difference in the bioactivities was indeed a result of their topological variations, ap-dG4 to p-rG4 interchange should result in the reversal of G4 function, hence transforming inactive ap-G4s to active p-rG4s.

**Figure 4.**
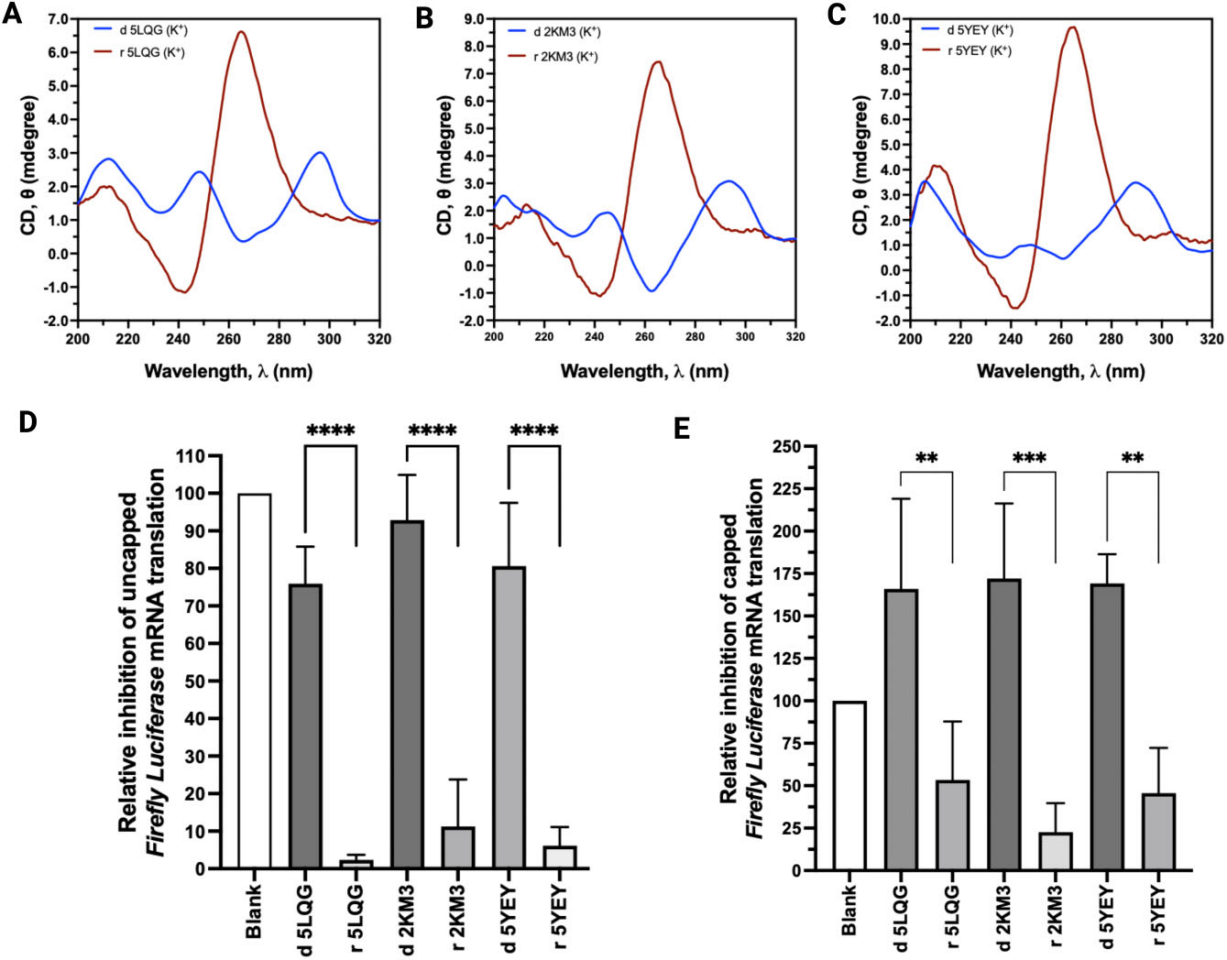
Conversion of nonbioactive G-rich ODNs to G-rich ONs not only changes their topologies but also rescues corresponding bioactivities. (**A**–**C**) CD spectra to demonstrate the change in topology of ap-G4-forming G-rich ODNs when they were converted into corresponding ONs (DNA→RNA). ODN/ON concentration was 10 μM, except for blank. (**D**) Relative impact of anti-parallel G-rich ODNs versus parallel G-rich ONs in the translation of uncapped FL mRNA in HEK293 cells-derived lysate. ODN/ON concentration was 10 μM, except for blank. (**E**) Relative impact of antiparallel G-rich ODNs versus parallel G-rich ONs in the translation of capped FL mRNA in HEK293 cells-derived lysate. ODN/ON concentration was 10 μM, except for blank. The bars represent the mean ± SEM of three independent biological experiments; statistical significance was tested by one-way ANOVA, multiple comparisons, and Dunnet correction: **P< .05, ***P< .0005, and ****P< .00005.

To test this hypothesis, we synthesized RNA counterparts of three ap-dG4s (namely, d 2KM3, d 5YEY, and d 5LQG). First, we confirmed their topological variations using CD spectroscopy, where we noticed expected topological switch as demonstrated in Fig. 4A (DNA→RNA conversion of the ap-G4 ODNs resulted in p-G4 ONs as evidenced by the CD peak switch from ∼295 nm for an ap-dG4 to ∼263 nm for a p-rG4). Next, we asked whether this topological switch in the G4 conformation is actually reflected in their bioactivity. We performed reporter mRNA translation experiments as described earlier. As demonstrated in Fig. 4B and C, ODN to ON conversion of G-rich sequences not only changes their structures (G4 topologies) but also inverses their biological functions—i.e. translationally inactive ap-dG4s are transitioned to translation repressing p-rG4s (Fig. 4D and E). Importantly, topology-dependent reversal of the action of G-rich ODNs is evident for both capped and uncapped FL mRNA translation. Additionally, we observed a dose-dependent response of p-G4 ODNs and ONs in the translation repression of FL mRNA (Supplementary Fig. S4).

### Only p-G4s directly interact with eIF4G1 via its HEAT1 domain

**Figure 5.**
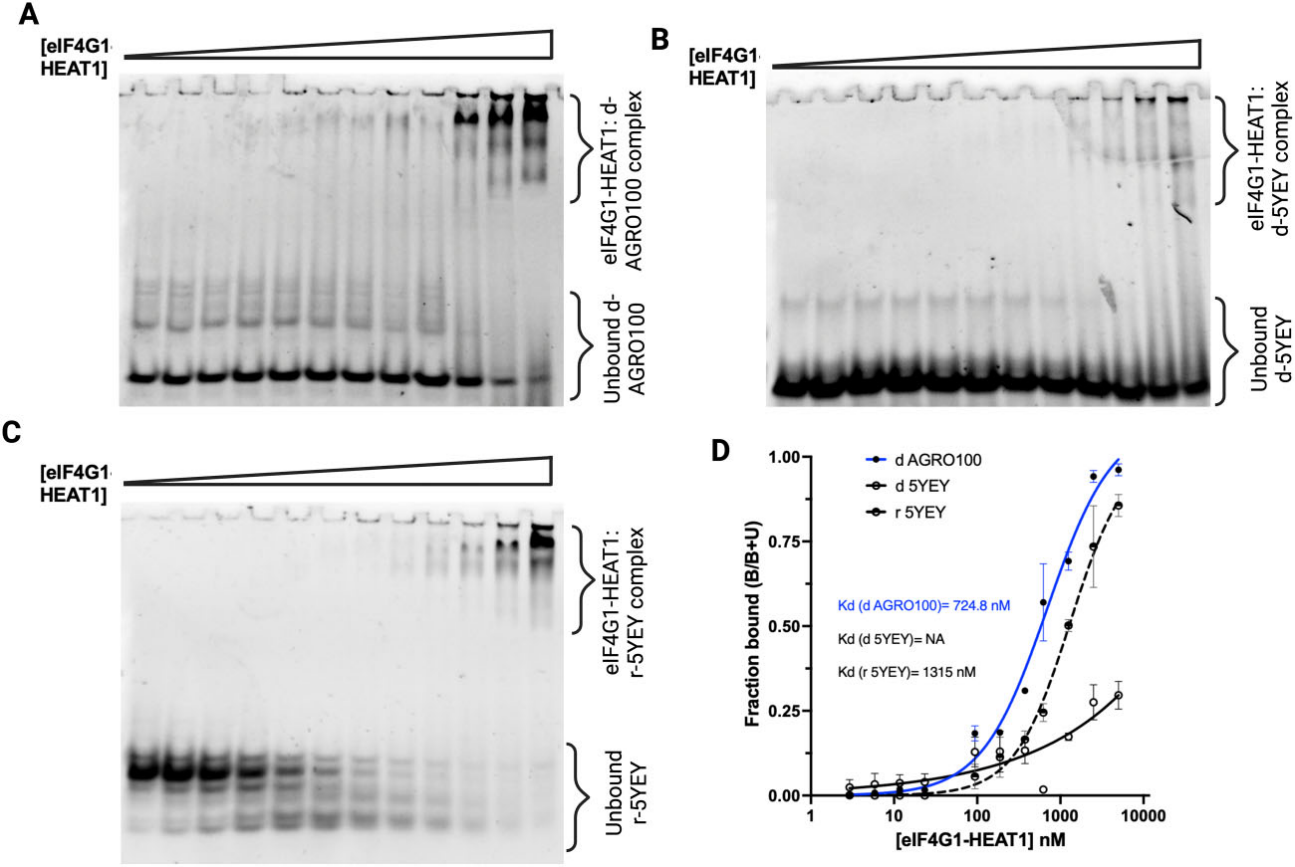
Anti-parallel G4 topology is detrimental for G4–eIF4G1 interaction. (**A**) Parallel-G4 forming AGRO100 binds with HEAT1 domain of eIF4G1 in a concentration-dependent manner. (**B**) However, ap-G4-forming 5YEY does not bind significantly with HEAT1. (**C**) The modification of d 5YEY to r 5YEY rescues 5YEY–HEAT1 binding, suggesting the need of parallel topology for the interaction of G4s with eIF4G1. (**D**) Nonlinear regression curve fitting for respective gel shifts (A–C) showed a tight binding between parallel d AGRO100 and HEAT1 (blue curve) with a *K*_d_ of 724.8 nM. The data represent average of three independent experiments. As evidenced from the gel shifts, there is no significant binding of ap-G4 with HEAT1 (solid black curve). Importantly, r 5YEY binds with HEAT1 with a *K*_d_ of 1.31 μM. For the gel images, protein concentration used was 5 μM in the rightmost lane and was serially diluted (1:1 ratio) moving from well 12 to well 2. First wells in each gel (left-most wells) contain no protein. More gel shift data with various p-G4s and ap-G4s are available in Supplementary Fig. S5.

We previously demonstrated that tetramolecular p-G4-forming tiRNAs can directly interact with eIF4G1 via its first HEAT domain (HEAT1) and displace eIF4G1 from the eIF4F complex, thereby inhibiting translation initiation [21]. Because of the observation that only parallel dG4 ODNs can repress eukaryotic mRNA translation, we reasoned that ap-G4s might interact with eIF4G1-HEAT1 differently. To test this hypothesis, we performed *in vitro* binding experiments of different G-rich ODNs with recombinant eIF4G1-HEAT1 and analyzed the resultant binding using a nondenaturing gel shift assay. We picked d AGRO100 as a p-G4 ODN candidate, and d 5YEY as an ap-G4 ODN candidate for these binding experiments (results of more candidates are shown in Supplementary Fig. S5). Interestingly, we observed that only the parallel dG4 (d AGRO100) binds to eIF4G-HEAT1 with significantly low *K*_d_ (724.8 nM) (Fig. 5A and D), while the anti-parallel dG4 (d 5YEY) did not bind to eIF4G-HEAT1 significantly at the given concentration as evidenced by a weaker gel shift and flatter binding curve for the latter (Fig. 5B and D). Importantly, when the topology of ap-dG4 (d 5YEY) was switched to p-rG4 (r 5YEY), the binding of G4–eIF4G1-HEAT1 was rescued (Fig. 5C and D).

## Discussion

Despite a plethora of studies investigating the potential of G-rich ODNs/ONs for their therapeutic applications [4–6, 22], we lack a clear mechanistic, biological, and structural understanding of why some G-rich ODNs/ONs behave differently than the others. In this report, using a series of biochemical, biophysical, and molecular biology techniques, we solved a part of the G-rich ODNs’ bioactivity puzzle. Our data made it clear that the translation inhibitory behavior of G-rich ODNs/ONs is mediated through their ability to fold into a particular topology G4 structure.

G-rich ODNs have been studied as molecules affecting cellular physiology [4]. They can be cytoprotective as well as cytotoxic based upon their sequence and length identities. While several of cytoeffective ODNs reported in the literature are G4-forming ODNs, how they impart the biological outcome *in trans* is unclear. The impact of G4s formed within both DNA and RNA molecules (e.g. telomeric sequences, 5′- and 3′-untranslated regions of mRNAs) *incis* has been widely studied. Such structures by themselves or in association with G4-binding proteins (G4BPs) were postulated to regulate almost every aspect of nucleic acid biogenesis, metabolism, and gene regulation [38–40]. However, it has been overlooked in the field why different *trans*-acting G4 ODNs/ONs show different and seemingly contrasting bioactivities (e.g. cytotoxic versus cytoprotective).

Motivated by our previous findings that G4-forming tetramolecular tiRNAs inhibit eukaryotic mRNA translation in the RRL-based system, we asked whether G-rich cytoeffective ODNs affect translation of mRNA reporters in the similar manner. Importantly, we used a custom-made human cell line extract-derived (HEK293 cells-derived) mRNA translation system instead of the commercially available RRL-based translation system [41] to minimize several unwanted non-physiological outcomes of the latter [42]. Specifically, G-rich ODNs, such as VEGF, AGRO100, CMYC, T40214, etc., repressed mRNA reporter translation to the levels similar of 5′TOG motif containing 5′tiRNA^Ala^ and its DNA analog, 5′tiDNA^Ala^ [7]. Importantly, the behavior of G-rich ODNs remains mostly similar on all the mRNA translation system settings we tested. However, the other class of G-rich ODNs came to our surprise as non-translation-repressing G-rich ODNs, such as Tel4, 2KM3, 5LQG, 5YEY, etc. By analyzing the sequence composition, it is obvious that it was not merely a G-richness of the sequences or the presence of poly-G tract on the 5′ end that affects their translation repression activity.

Our data strongly suggest that topology of G4-forming ODNs is one of the key determinants of their activities, at least in their ability to affect mRNA translation. Since protein synthesis is the central process that is tightly regulated during specific physiological conditions such as changing environment or stress, an influence on the efficiency of mRNA translation by specific ODNs can have direct consequences for cellular metabolism and, thus, cell death or survival. One of the examples of such influence is the ability of specific rG4s to promote formation of stress granules (SGs), dynamic pro-survival RNA granules that help cell coping with stressful environment [43, 44]. Whether ODN cytotoxicity/cytoprotection correlates with its abilities to engage in the stress-responsive or pro-apoptotic programs remains to be determined in future.

G4 structures have been previously shown to interact with several G4BPs, thereby influencing downstream cellular processes [34, 40, 45]. We and others previously discovered that, once transfected in the cells, G4 ODNs/ONs can interact with cellular stress response-associated proteins such as G3BP1, YBX1, TIA1, and eIF4G1, and can induce SG assembly formation and translation repression [7, 21, 32–34, 46]. However, our findings in this report revealed that translation suppression ability of G4s is not universal, rather it is topology dependent. In addition, we previously showed that the mechanism of mRNA translation inhibition mediated by the rG4s involved the displacement of protein factors engaged in translation initiation [7, 21, 33]. Consequently, we reasoned that the bioactive ODN G4s may also directly interact with the components of the mammalian translational machinery and/or protein synthesis regulators, as reported for G4-assembling tiRNAs [7, 21, 47]. Indeed, only p-G4s can directly interact with the HEAT1 domain of eIF4G1 protein, thereby disassembling the eIF4F translation initiation complex. Contrarily, the ap-G4s do not interact with eIF4G1-HEAT1 domain and hence do not show translation inhibitory effects. A HEAT repeat is a tandem repeat structural motif in multiple proteins. It is composed of two alpha-helices linked by a short loop (the name “HEAT” is derived from the combination of letters of four protein names in which this repeat structure was first described, namely **H**untingtin, **E**longation factor 3, the regulatory **A** subunit of protein phosphatase 2A, and the mechanistic **T**arget of rapamycin 1) [48]. In addition to being present in EIF4G1 and these four proteins, HEAT repeat motifs are found in various other cellular proteins such as the nuclear pore transport protein importin β, Ro autoantigen, and splicing factor SAP155. At the functional level, HEAT motif repeat-containing structures are involved in a multitude of cellular processes, including intracellular transport, signaling, and protein synthesis by facilitating protein–protein and protein–nucleic acid interactions [49–52]. While the amino acid composition conservation among HEAT domains is weak, their structural conservation is high. Our current finding that G-rich ODNs can interact with the HEAT1 motif of EIF4G1 also suggests that they can selectively interact with other HEAT-containing proteins, thus potentially influencing multiple cellular processes.

In summary, we identified the molecular mechanism by which G-rich ODNs can modulate different biological effects. We discovered that G4s can have totally distinct biological outcomes depending from their topological differences. Specifically, G4 binding to HEAT1 domain of eIF4G1 can clearly distinguish between a parallel versus anti-parallel topology G4 and result in completely different functional consequences. Our findings further contribute to the understanding of mechanistic differences created by topological variabilities within the G4-forming therapeutic ODNs.

## Supplementary Material

gkaf590_Supplemental_Files

## Data Availability

All data are incorporated into the article and its online supplementary material.

## Notes

Present address: Department of Biochemistry and Molecular Biology, University of Kansas Medical Center, Kansas City, KS 66160, United States
