# Supplementary material for: G-quadruplex topologies determine the functional outcome of guanine-rich bioactive oligonucleotides
Source: Nucleic Acids Res. 2025 Jun 28;53(12):gkaf590. doi: 10.1093/nar/gkaf590 (PMC12205368; doi:10.1093/nar/gkaf590)
Supplement: gkaf590_Supplemental_Files [file gkaf590_supplemental_files.zip › Parallel vs anti parallel G-quadruplexes_NAR_Supp_resubmission-FINAL.pdf]

Supplementary material for

G-quadruplex topologies determine the functional outcome of guanine rich bioactive oligonucleotides

Prakash Kharel[1*], Nupur Bhatter[1], Safiyah Zubair[1], Shawn Lyons[2], Paul J. Anderson[1], and Pavel Ivanov[1*]

[1]Division of Rheumatology, Inflammation, and Immunity, Department of Medicine, Brigham and Women's Hospital, Harvard Medical School, Boston, MA 02115, USA.

[2]Department of Biochemistry and Cell Biology, School of Medicine, Boston University, Boston, MA 02118, USA.

*Correspondence
pkharel@bwh.harvard.edu (P. Kharel)
pivanov@bwh.harvard.edu (P. Ivanov)

Results

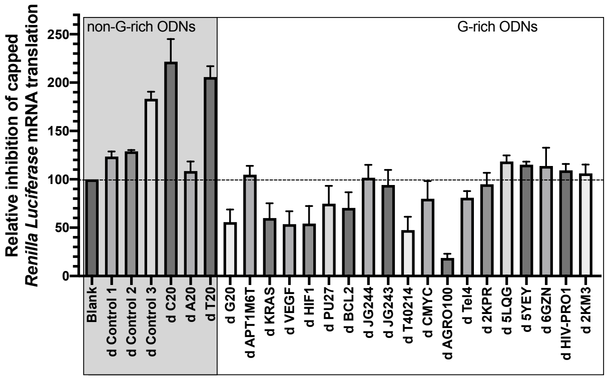

Figure S1. Impact of different G-rich ODNs in the translation of capped *Renilla luciferase* mRNA in HEK293 cells derived lysate. The bars represent the mean + /- SEM of three independent biological experiments.

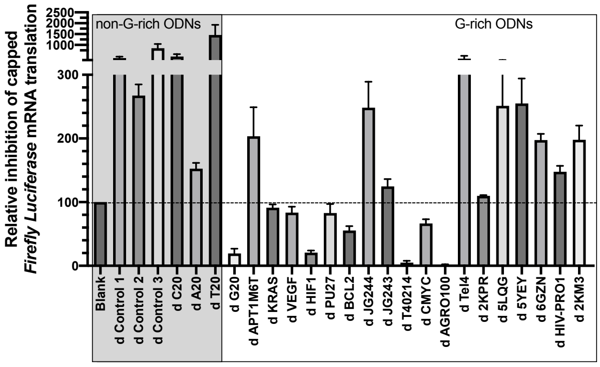

Figure S2. Impact of different G-rich ODNs in the translation of capped *Firefly luciferase* mRNA in HEK293 cells derived lysate. The bars represent the mean + /- SEM of three independent biological experiments.

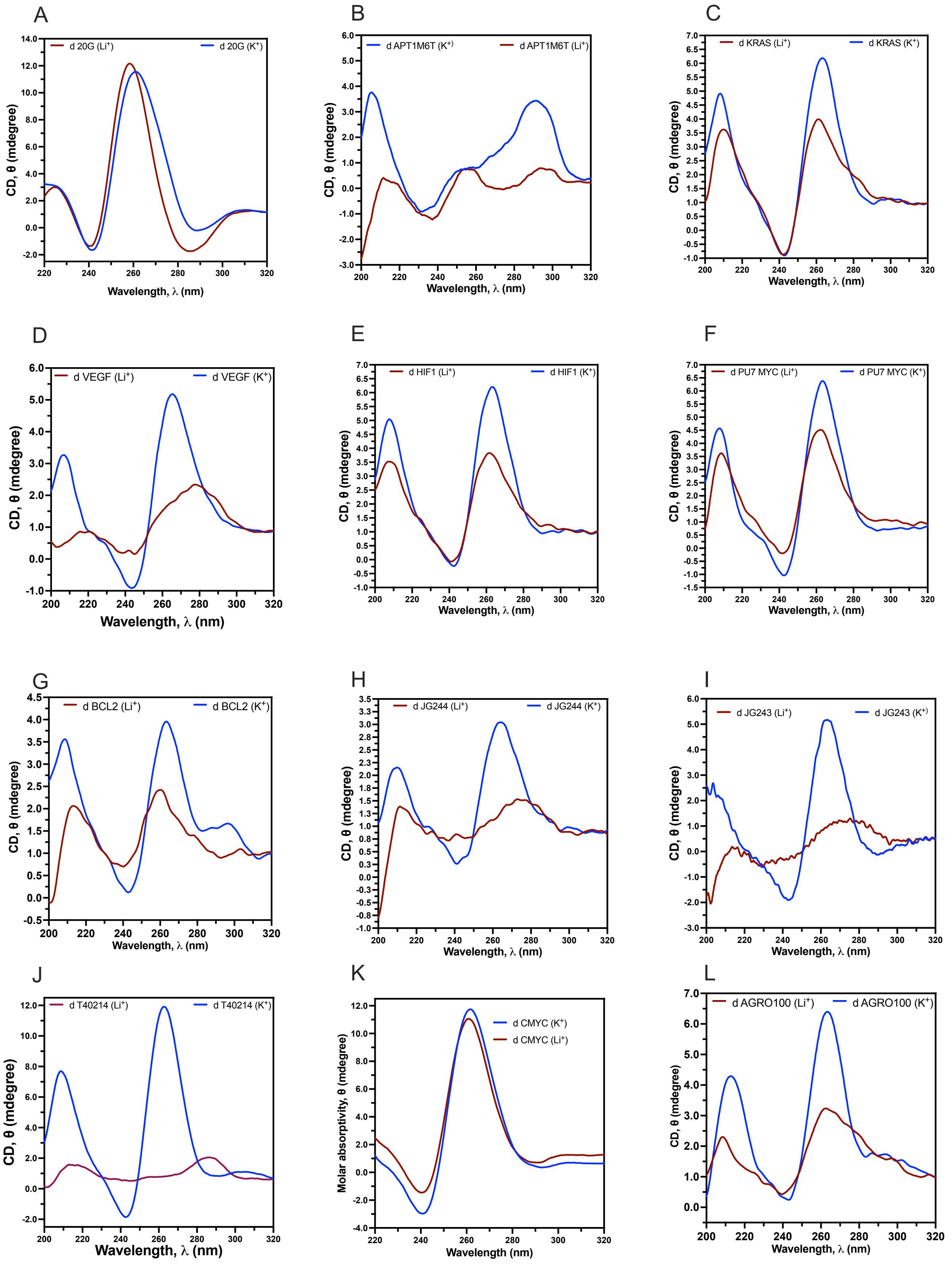

Figure S3 Continued to the next page……

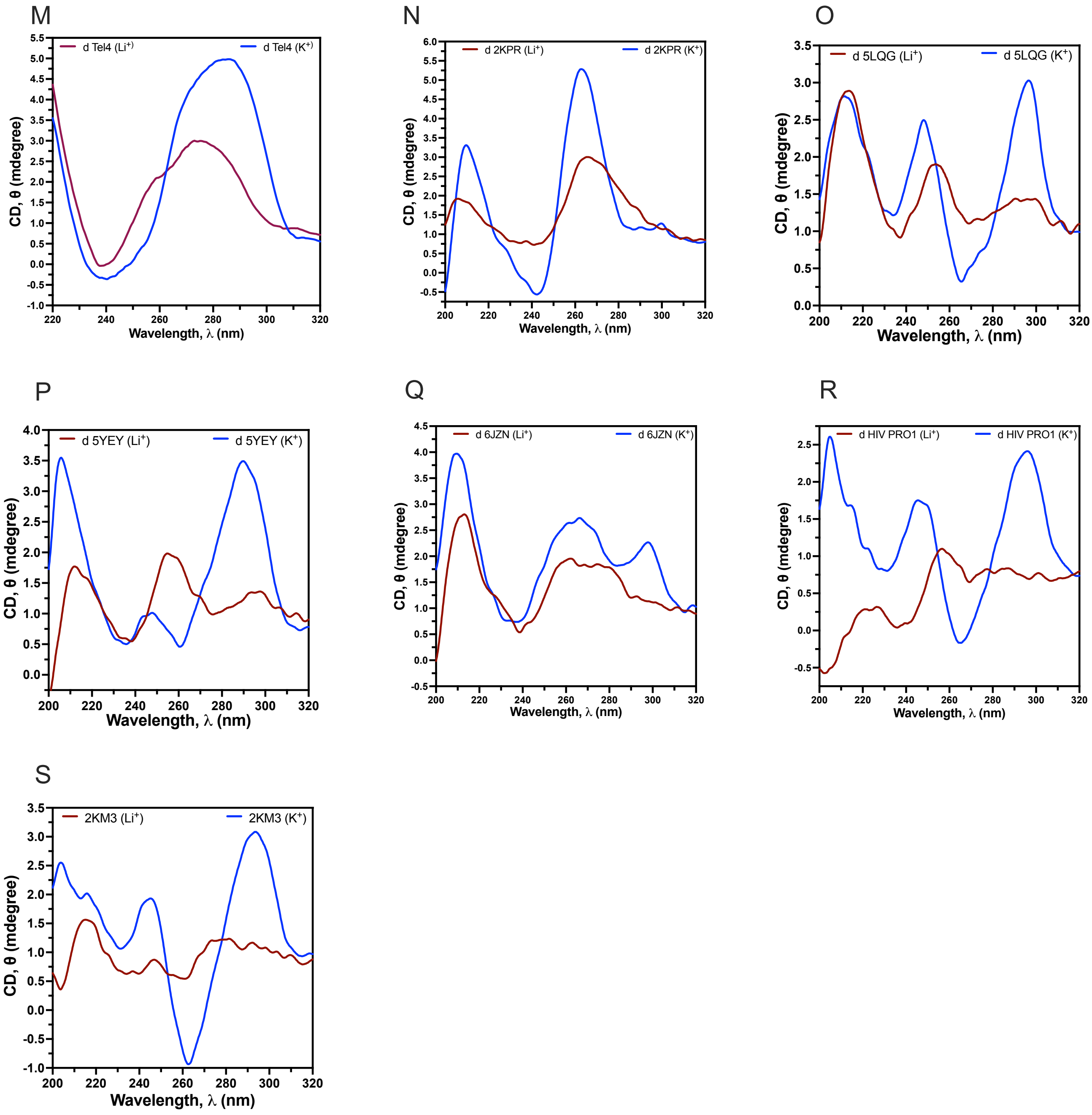

Figure S3. CD spectra of the Complete set of G-rich ODNs used in this study, topology is assigned based on these data plus information extracted from published data (full reference provided in associated supplementary table 1). (**A**) d G20: parallel topology G4, (**B**) d APT1M6T: anti-parallel topology G4, (**C**) d KRAS: parallel topology G4, (**D**) d VEGF: parallel topology G4 (**E**) HIF1: parallel topology G4, (**F**) d pu7 MYC: parallel topology G4, (**G**) d BCL2: predominately parallel G4, (**H**) d JG244: parallel G4, (**I**) d JG243: parallel G4, (**J**) d T40214: parallel G4, (**K**) d CMYC: parallel G4, (**L**) d AGRO100: multimeric predominately parallel G4, (**M**) d Tel4: anti-parallel G4, (**N**) d 2KPR: hybrid G4, (**O**) d 5LQG: anti-parallel G4, (**P**) d 5YEY: anti-parallel G4, (**Q**) d 6JZN: hybrid/anti-parallel G4, (**R**) d HIV PRO1: anti-parallel G4, and (**S**) d 2KM3: anti-parallel G4.

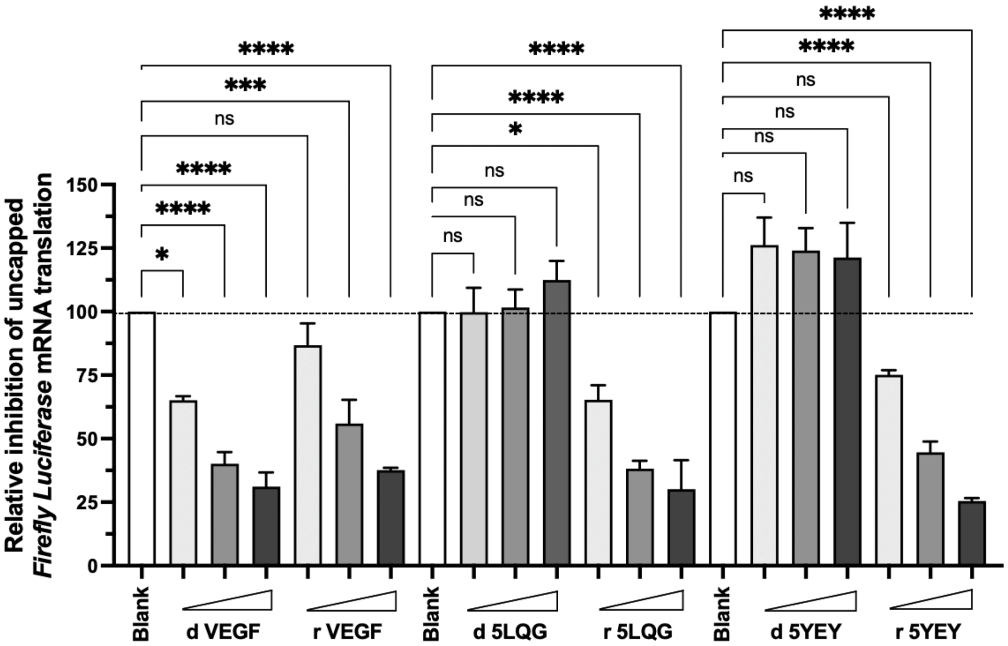

Figure S4. Concentration dependent effect of bioactive G-rich ONs in the translation of uncapped *Firefly luciferase* translation (in contrast to the inactivity of corresponding ODNs). The bars represent the mean + /- SEM of three independent biological experiments, statistical significance was tested by One-way ANOVA using water treatment as a blank control, Multiple comparisons, Dunnet correction, and p-values indicated only where there is a significant repression. * p<0.5, ** p<0.05, *** p<0.0005, and **** p<0.00005.

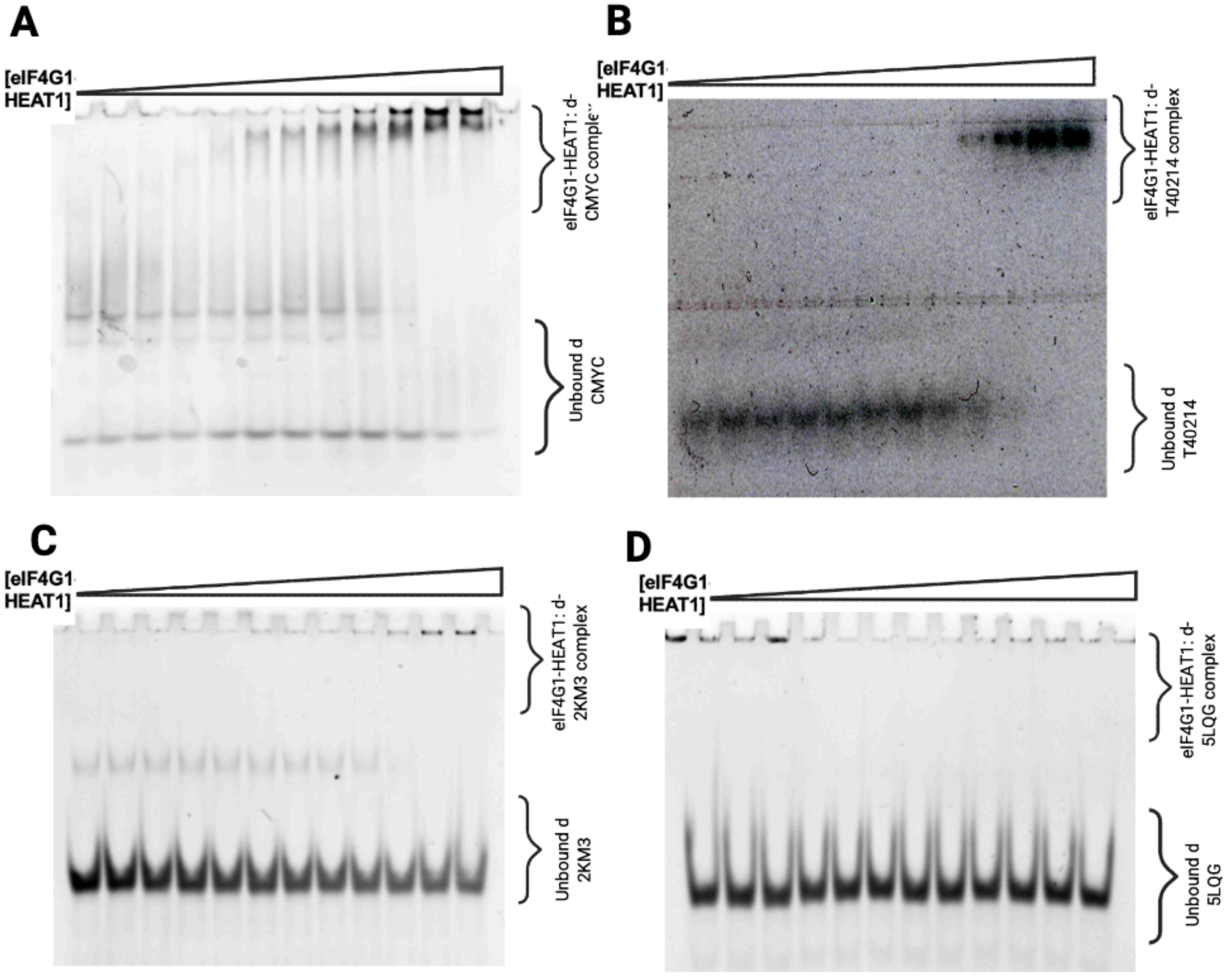

Figure S5. Anti-parallel G4 topology is detrimental in G4-eIF4G1 interaction (Extension of Figure 5). (**A**) Parallel G4 forming d CMYC binds with HEAT1 domain of eIF4G1 in concentration dependent manner. (**B**) Parallel G4 forming d T40214 binds to HEAT1 domain of eIF4G1 in concentration dependent manner, in this particular case, d T40214 ODN was 5' end-radiolabelled with ATP, [γ-32P]-3000 Ci/mmol (Revvity) using T4 Polynucleotide Kinase (New England Biolabs). Labelled ODN was purified by gel filtration with Illustra MicroSpin G-25 Columns (GE Healthcare), and binding experiment was performed as described for other ODNs. The dry gel was exposed to X-ray film at dark and imaged. (**C**) Anti-parallel G4 forming d 2KM3 does not bind significantly with HEAT1, and (**D**). Anti-parallel G4 forming d 5LQG does not bind significantly with HEAT1.
